# Microbial eukaryote diversity in the marine oxygen minimum zone off northern Chile

**DOI:** 10.3389/fmicb.2014.00543

**Published:** 2014-10-28

**Authors:** Darren J. Parris, Sangita Ganesh, Virginia P. Edgcomb, Edward F. DeLong, Frank J. Stewart

**Affiliations:** ^1^School of Biology, Georgia Institute of TechnologyAtlanta, GA, USA; ^2^Woods Hole Oceanographic Institution, Woods HoleMA, USA; ^3^Department of Civil and Environmental Engineering, Massachusetts Institute of Technology, Parsons Laboratory 48Cambridge, UK; ^4^Center for Microbial Ecology, Research and EducationHawaii, HI, USA

**Keywords:** microeukaryote, ETSP OMZ, diversity, 18S, low oxygen

## Abstract

Molecular surveys are revealing diverse eukaryotic assemblages in oxygen-limited ocean waters. These communities may play pivotal ecological roles through autotrophy, feeding, and a wide range of symbiotic associations with prokaryotes. We used 18S rRNA gene sequencing to provide the first snapshot of pelagic microeukaryotic community structure in two cellular size fractions (0.2–1.6 μm, >1.6 μm) from seven depths through the anoxic oxygen minimum zone (OMZ) off northern Chile. Sequencing of >154,000 amplicons revealed contrasting patterns of phylogenetic diversity across size fractions and depths. Protist and total eukaryote diversity in the >1.6 μm fraction peaked at the chlorophyll maximum in the upper photic zone before declining by ~50% in the OMZ. In contrast, diversity in the 0.2–1.6 μm fraction, though also elevated in the upper photic zone, increased four-fold from the lower oxycline to a maximum at the anoxic OMZ core. Dinoflagellates of the Dinophyceae and endosymbiotic Syndiniales clades dominated the protist assemblage at all depths (~40–70% of sequences). Other protist groups varied with depth, with the anoxic zone community of the larger size fraction enriched in euglenozoan flagellates and acantharean radiolarians (up to 18 and 40% of all sequences, respectively). The OMZ 0.2–1.6 μm fraction was dominated (11–99%) by Syndiniales, which exhibited depth-specific variation in composition and total richness despite uniform oxygen conditions. Metazoan sequences, though confined primarily to the 1.6 μm fraction above the OMZ, were also detected within the anoxic zone where groups such as copepods increased in abundance relative to the oxycline and upper OMZ. These data, compared to those from other low-oxygen sites, reveal variation in OMZ microeukaryote composition, helping to identify clades with potential adaptations to oxygen-depletion.

## Introduction

Marine oxygen minimum zones (OMZs) support complex microbial communities adapted for life under low oxygen conditions. In the anoxic OMZ of the Eastern Tropical South Pacific (ETSP) off Chile and Peru, aerobic respiration of sinking organic matter combines with a decline in water column mixing to deplete dissolved oxygen to below detection (<15 nM; Thamdrup et al., 2012). Oxygen depletion at the ETSP OMZ core (~100–400 m) selects for communities dominated by bacteria and archaea (prokaryotes) capable of anaerobic or microaerophilic metabolism (Stevens and Ulloa, 2008; Ulloa et al., 2012). These microorganisms make important contributions to marine nitrogen, sulfur, and carbon cycling (Wright et al., 2012), for example as mediators of nitrogen loss through denitrification and anaerobic ammonium oxidation (Thamdrup et al., 2006; Lam et al., 2009). Although most large multicellular eukaryotes are absent from anoxic marine waters, severely oxygen-depleted layers in other OMZ regions contain a diverse community of microbial eukaryotes (microeukaryotes) including protists and fungi, as well as zooplankton with potential adaptations to oxygen limitation (Orsi et al., 2012; Orsi and Edgcomb, 2013; Teuber et al., 2013). As suggested for a coastal OMZ (Orsi et al., 2012), microeukaryotes in the ETSP OMZ may play important ecological roles, for example through predation or symbiotic interactions with prokaryotes. However, while bacteria and archaea in the ETSP OMZ are becoming better understood, and numerous studies have examined benthic meiofaunal diversity beneath OMZs (Neira et al., 2001; Levin, 2003), little is known about pelagic eukaryote diversity in this unique oxygen-depleted environment.

Marine microeukaryotes and zooplankton can significantly affect the structure and function of prokaryote communities and are now recognized as pivotal members of aquatic food webs in numerical models of nutrient cycling and in paradigms of surface and deep-ocean ecology (Arístegui et al., 2009). Protists impact major nutrient cycles directly and indirectly, through grazing on prokaryotic prey and consequent regeneration of nutrients, and through direct modification of organic matter (sinking particulate and dissolved) (Taylor, 1982; Sherr and Sherr, 2002; Tang et al., 2010). Zooplankton surfaces, guts, and fecal pellets are habitats for bacterial growth (Tang et al., 2010), supporting local cell densities orders of magnitude higher than in the surrounding water (Grossart et al., 2003). Studies in oxygen deficient waters of the North Pacific and the anoxic Cariaco Basin have revealed unique protist communities with diversity and abundances varying along gradients of oxygen, sulfide, and methane concentration (Taylor et al., 2001; Li et al., 2008; Lin et al., 2008; Edgcomb et al., 2011a; Orsi et al., 2011, 2012). Anaerobic and microaerophilic species of ciliates and euglenid flagellates seem to be enriched in anoxic water layers (Lynn, 2008; Edgcomb et al., 2011b; Orsi et al., 2011, 2012). Recent studies of marine sediments suggest that some euglenids engage in symbiosis with nitrate reducing, sulfur oxidizing, epibiotic bacteria (Bernhard et al., 2000; Yubuki et al., 2009; Edgcomb et al., 2010). Cryptic symbioses between ciliates and methanogenic archaea have been suggested to possibly contribute significantly to methanogenesis in OMZs (Orsi et al., 2012). Furthermore, OMZs are known to harbor larger zooplankton such as copepods with specific ecophysiological and behavioral adaptations for persistence under low oxygen (e.g., reduced overall metabolic rates, enhanced activity of anaerobic pathways, diel migrations) (Childress and Seibel, 1998; Teuber et al., 2013). However, interactions of OMZ zooplankton with other community members are not well understood.

In some ocean regions, picoeukaryotes smaller than 3 μm may be among the most abundant cells in the water column (Moon-van der Staay et al., 2001; Massana et al., 2011), playing roles in primary production, food web dynamics, and mineral cycling (Fogg, 1995; Diez et al., 2001; Moon-van der Staay et al., 2001; Worden et al., 2004). However, many of these smaller microeukaryotes are new to science, and we are just beginning to understand their ecology (Moon-van der Staay et al., 2001; Worden et al., 2004). Because of their small size, picoeukaryotes may be overlooked in gene surveys focusing only on larger particulate size fractions of filtered water samples (>3.0 μm). Indeed, no studies of OMZs have presented a size-fractionated comparison of eukaryotic community structure. Characterizing microeukaryote diversity and distribution among different OMZ regions and size fractions is a necessary step toward quantifying the ecological importance of these organisms for bulk chemical cycling and eukaryote-prokaryote interactions in OMZs and for clarifying the effects of severe oxygen depletion on planktonic community structure.

Here, we use 18S rRNA gene amplicon sequencing to provide a snapshot of eukaryote community structure in two size fractions (0.2–1.6 μm, >1.6 μm) along a depth gradient through the ETSP OMZ off northern Chile. To our knowledge, this is the first community-level genetic survey of microbial eukaryotes in this anoxic water column. We highlight differences between the two size fractions, across the OMZ oxygen gradient, and make comparisons to 18S gene surveys from other marine environments.

## Materials and methods

### Sample collection and DNA extraction

Microbial community samples were collected from the ETSP OMZ as part of the Center for Microbial Ecology: Research and Education (C-MORE) BiG RAPA (Biogeochemical Gradients: Role in Arranging Planktonic Assemblages) cruise aboard the *R/V Melville* (18 November—14 December, 2010). Seawater was sampled from seven depths spanning the oxic photic zone and oxycline (5 and 32 m samples), the suboxic (O_2_ < 10 μm) upper OMZ (70 m), the anoxic OMZ core (110, 200, 320 m), and the oxic zone beneath the OMZ (1000 m) from a single site (20° 05.0 S, 70° 48.0 W) off the coast of Iquique, Chile on November 19th (5 m), 20th (32 m), 21st (70, 110, 200, 320 m) and 23rd (1000 m). Water was sampled from Niskin bottles on a rosette containing a CTD profiler (Sea-Bird SBE 911plus) with a dissolved oxygen sensor and fluorometer. A peristaltic pump was used to filter water (10 L) through an in-line prefilter (GF/A, 1.6 μm pore-size, 47 mm dia., Whatman) to retain larger eukaryotes and a Sterivex™ filter (0.22 μm pore-size, Millipore) to retain picoeukaryotes. Prefilters and Sterivex™ filters were saturated with lysis buffer (~1.8 ml; 50 mM Tris-HCl, 40 mM EDTA, and 0.73 M Sucrose) and frozen until analysis.

Genomic DNA was extracted as described in Ganesh et al. (2014). Cells were lysed by adding lysozyme (2 mg in 40 μl of Lysis buffer per filter) directly to a microcentrifuge tube containing a pre-filter or to the Sterivex™ cartridge, sealing the caps/ends, and incubating for 45 min at 37°C. Proteinase K (1 mg in 100 μl lysis buffer, with 100 μl 20% SDS) was added and the tubes further incubated for 2 h at 55°C. Lysate was removed and DNA was extracted once with Phenol:Chloroform:Isoamyl Alcohol (25:24:1) and once with Chloroform:Isoamyl Alcohol (24:1). The purified aqueous phase was concentrated using Amicon Ultra-4 w/100 kDa MWCO centrifugal filters. Purified DNA aliquots from each depth and size fraction were used for PCR.

### 18S rRNA gene PCR and amplicon sequencing

A 450-bp fragment spanning the variable V4 region of the 18S rRNA gene was PCR amplified using the universal forward primer 3NDF and the universal reverse primer V4_euk_R1 (Bråte et al., 2010). Primers were appended with Roche 454 sequencing adaptors and sample-specific barcodes as described in the protocol established for the Human Microbiome Project by the Broad Institute (Jumpstart Consortium Human Microbiome Project Data Generation Working Group, 2012). Reactions (25 μl) were performed with 50–150 ng template DNA in Platinum PCR Mastermix (Invitrogen) and run on an Eppendorf Mastercyler thermocycler. The PCR program for amplification was: 94°C for 2 min, followed by 34 cycles of 30 s at 94°C, 30 s at 59°C, 60 s at 72°C with a final extension at 72°C for 7 min. Amplicons were purified using the QIAQuick PCR Clean-Up Kit, quantified on a Qubit® 2.0 Fluorometer, and pooled at equimolar concentrations. Pooled, barcoded amplicons were sequenced unidirectionally using the Roche Genome Sequencer FLX Instrument with Titanium chemistry. All sequence data generated in this study will be made publicly available in the NCBI Sequence Read Archive and is accessible under BioProject ID number PRJNA263803.

### Data analysis

Amplicons were analyzed according to established protocols using the QIIME package (Caporaso et al., 2010). Barcoded 18S datasets were de-multiplexed and filtered to remove low quality sequences using default parameters (minimum quality score = 25, minimum sequence length = 200, no ambiguous bases allowed). De-multiplexed sequences were clustered into Operational Taxonomic Units (OTUs) at 97% sequence similarity, with taxonomy assigned to representative OTUs from each cluster by queries against the Protist Ribosomal Reference database, PR2 (as of March 2014, Guillou et al., 2012). OTU counts were rarefied and alpha diversity was quantified at a uniform sequencing depth based on the lowest sequence count from the protist only subset at 70 m (*N* = 1308 sequences) using the phylogenetic diversity (PD) metric (Faith, 1992). To evaluate differences in community composition between samples, sequences were aligned using the PyNAST aligner in QIIME and beta diversity was calculated using the weighted Unifrac metric, which compares samples based on the phylogenetic relatedness (branch lengths) of OTUs in a community, taking into account relative OTU abundance (Lozupone and Knight, 2005). Sample relatedness based on Unifrac was visualized using a two-dimensional Principal Coordinate Analysis.

## Results

### Oxygen

**Figure 1:**
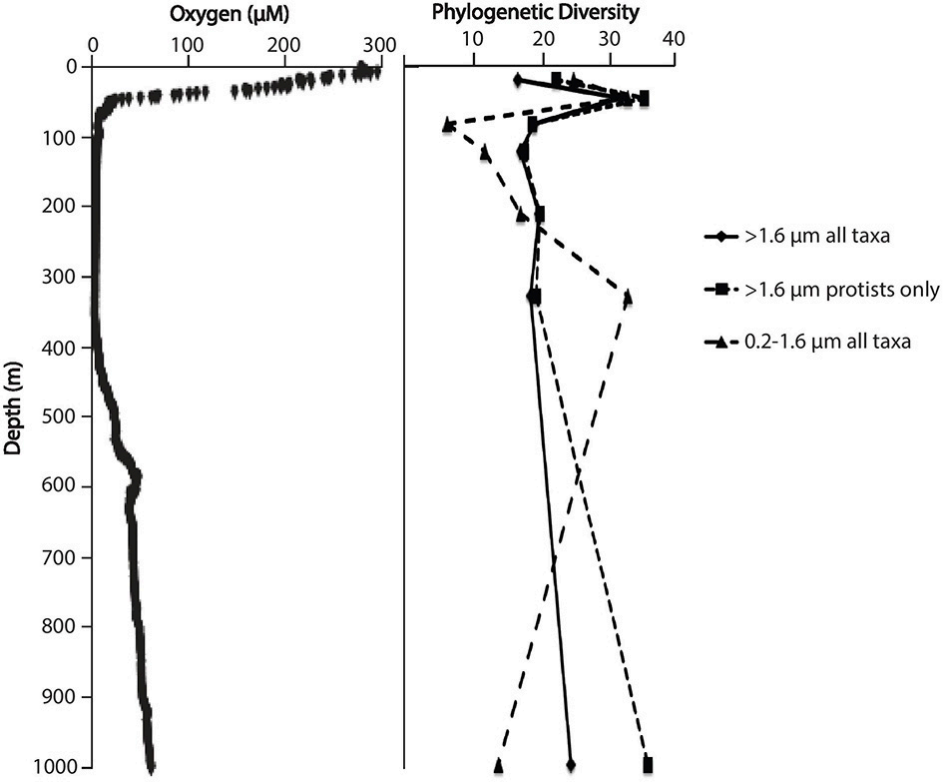
**Dissolved oxygen concentration and phylogenetic diversity as a function of water column depth**. Diversity data points are mean values based on rarefaction of OTU counts at a standardized sequence count (*n* = 1308 per sample).

As reported previously (Ganesh et al., 2014), the sample site was characterized by steep vertical gradients in dissolved oxygen (Figure 1). Oxygen concentrations measured with the SBE sensor ranged from ~250 μM at the surface to below 5 μM through the OMZ core (~100–400 m), before increasing below 400 m to ~60 μM at 1000 m. Prior measurements using high sensitivity switchable trace oxygen sensors with resolution in the nanomolar range indicate that the ETSP OMZ core is anoxic, with oxygen concentrations below 30 nM (Revsbech et al., 2009; Thamdrup et al., 2012). The base of the photic zone (1% surface PAR) occurred at ~40 m, with chlorophyll concentration peaking at 32 m (1.97 μg l^−1^).

### Phylogenetic diversity

Following removal of low quality reads, 44,975 and 109,141 sequences representing both protist and metazoan taxa were analyzed to assess diversity in the 0.2–1.6 μm (picoeukaryote) and >1.6 μm size fractions, respectively (median per sample: 6760 and 15,564 for small and large size fractions; Table 1, Figure 1). In the >1.6 μm fraction, phylogenetic diversity (PD), which measures the total branch length connecting all OTUs in the 18S rRNA gene phylogeny, peaked at 32 m within the primary chlorophyll maximum, but was comparable between surface (5 m) and anoxic (110–320 m) depths (Figure 1). In contrast, PD of the picoeukaryote fraction was bimodal with depth, peaking first within the chlorophyll maximum layer, declining to a minimum along the oxycline, and then progressively increasing four-fold within the OMZ to peak again at 300 m, where diversity was equivalent to that observed at 32 m and almost two-fold higher than that of the >1.6 μm fraction at the same depth (Figure 1).

**Table 1:** **18S rRNA gene amplicon sequencing statistics**.

**Sample**	**Count**	**OTU^1^**
TOTAL sequences	196,027^2^	
Mean length	410 bp	
TOTAL unique OTUs		2331
5 m 0.2–1.6 μm	4679	463
5 m >1.6 μm	26,127	639
32 m 0.2–1.6 μm	6760	620
32 m >1.6 μm	12,943	946
70 m 0.2–1.6 μm	2036	47
70 m >1.6 μm	15,564	462
110 m 0.2–1.6 μm	8097	205
110 m >1.6 μm	14,868	440
200 m 0.2–1.6 μm	5633	270
200 m >1.6 μm	13,388	504
320 m 0.2–1.6 μm	7148	692
320 m >1.6 μm	9562	382
1000 m 0.2–1.6 μm	10,622	310
1000 m >1.6 μm	16,689	660

1 *Observed number of operational taxonomic units (>97% nucleotide similarity)*.

2 *Total sequences before quality filtering*.

### Community composition

**Figure 2:**
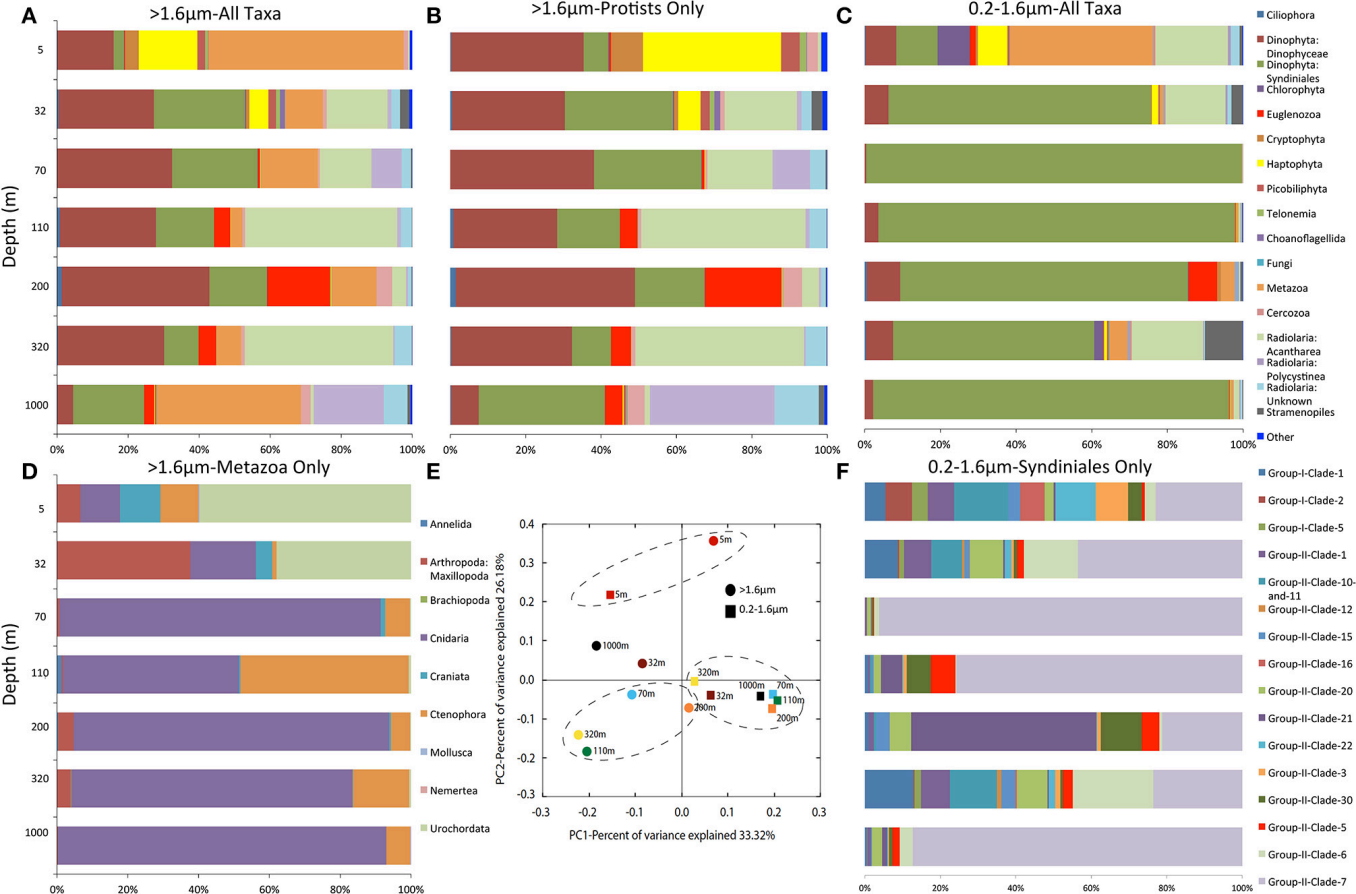
**Community taxonomic composition in the **(A)** >1.6 μm fraction based on all 18S rRNA gene sequences**. **(B)** >1.6 μm fraction with metazoan sequences excluded. **(C)** 0.2–1.6 μm fraction based on all sequences. **(D)** >1.6 μm fraction based only on sequences matching metazoa. **(E)** shows a principle component analysis of community taxonomic relatedness based on all sequences from both size fractions, as quantified by the weighted Unifrac metric. Depths within the OMZ are circled. **(F)** 0.2–1.6 μm size fraction based only on sequences matching Syndiniales dinoflagellates.

Eukaryote community composition differed substantially along the OMZ oxygen gradient and between size fractions (Figure 2). Based on weighted Unifrac distances, sequences of the >1.6 μm fraction were less similar across depths compared to the 0.2–1.6 μm fraction (Figure 2E), reflecting a broader range of major taxonomic groups in the larger fraction. Indeed, excluding the 5 m surface sample, samples of the 0.2–1.6 μm fraction were dominated by a single Order (Syndiniales). The >1.6 μm fraction from the oxic sub-OMZ (1000 m) and both size fractions from the surface (5 m) sample were relative outliers, clustering apart from those of the oxycline and OMZ depths (Figure 2E).

The separation of the surface samples was due partly to their enrichment in metazoan and haptophyte sequences (45–72% of total, compared to 3–13% for sub-surface depths; Figure 2C). The diversity of the metazoan pool peaked at 5 m (Figure 2D), with groups such as the Appendicularia (Urochordata) and arthropods over-represented relative to OMZ depths (Figure 2D). In contrast, cnidarians and ctenophores comprised the bulk of the metazoan sequences in the larger size fraction within and below the OMZ. Sequences matching copepods (Maxillopoda) declined in relative abundance from the primary chlorophyll layer (32 m) to the upper OMZ, but increased again to a local maximum at the OMZ core (200, 320 m; Figure 2D).

Protists were abundant (45–96%) at all depths but comprised the vast majority (>85% of total) of the eukaryote community within the oxycline and OMZ (Figure 2B). Several groups followed clear depth-specific trends. Euglenozoan flagellates of the class Diplonemea represented a major portion of the OMZ community, comprising 18% of total sequences from the larger size fraction at 200 m, but were almost absent (<2%) from oxycline and surface communities. Ciliate sequences related to the Litostomatea were only observed in the 110 m and 200 m OMZ depths, although in low abundance (0.5–1.5% of all sequences). Photosynthetic algae of the Cryptophyta, Haptophyta, Picobiliphyta, Choanoflagellida, and Stramenopiles (diatoms) were confined primarily to the surface, representing ~20% of the sequence pool from the larger size fraction at 5 m (Figure 2B). In contrast, radiolarians in this fraction became progressively more dominant with depth, with unidentified members of the Acantharea peaking at the upper and lower OMZ boundaries (110 and 320 m), accounting for 42 and 41% of sequences, respectively, and Polycistenea radiolarians peaking at over 1/3 of protist sequences at 1000 m beneath the OMZ (Figure 2B).

The most consistently abundant protist OTUs were similar to uncultured Dinophyceae and the predominantly parasitic dinoflagellate taxon Syndiniales. These groups were detected at all depths, representing 42–68% of all protist sequences in the larger size fraction. Aside from a decline in Dinophyceae representation at 1000 m (to ~7%), sequence abundance within Dinophyceae and Syndiniales in the larger size fraction did not differ notably between oxic and OMZ depths (Figure 2B). In contrast, the picoeukaryotic fraction (excluding that of the surface sample) was dominated by Syndiniales dinoflagellates (53–99% of total), which showed substantial depth-specific variation in clade diversity across depths (Figure 2F). Syndiniales diversity peaked at the surface and again within the OMZ, with Group II clades 5, 21, and 30 enriched at the OMZ depths (Figure 2E) and Group II clade 7 being the most abundant group at all other depths.

## Discussion

As observed in other low oxygen marine waters (Table 2), the ETSP OMZ harbors a diverse community of microeukaryotes with complex patterns of taxonomic structuring across oxygen gradients and between cellular size fractions. Phylogenetic diversity of eukaryotes in the >1.6 um size fraction, which presumably captures the majority of eukaryotic biomass, peaked in the oxic, photic zone in the layer of maximum chlorophyll fluorescence. This pattern is consistent with the hypothesis that resource availability supports a greater number of niches for microeukaryotes. Enhanced diversity in the photic zone may also be driven by a greater overall abundance in near-surface waters of larger organisms (metazoa) acting as hosts for symbiotic or parasitic protists. Interestingly, diversity within the larger size fraction from anoxic OMZ depths was equivalent to that at the surface (5 m) and in the oxic bottom water. The overall diversity trend in the larger size fraction did not vary substantially when only protists were analyzed (Figure 1), indicating that metazoan sequences had negligible effects on diversity calculations.

**Table 2:** **Relative abundance of dominant taxa across diverse low oxygen sites**.

**Site**	**Oxygen^a^**	**Taxonomic group^b^**	**Relative%**	**References**	**Method^c^**	**Sequences**
North Pacific Ocean Oxic site, January sampling	Oxic at 5 m	Ciliophora	20	Countway et al., 2010	CS	524
		Unknown alveolate	20			
		Dinophysceae	16			
	Oxic at 150 m	Polycistenea	70			
		Marine Alveolate Group I	12			
		Acantharea	3			
	Oxic at 500 m	Polycystenea	55			
		Marine Alveolate Group II	23			
		Marine Alveolate Group I	7			
Hamelin Pool, Shark Bay microbialites Permanent anoxic basin	**Anoxic 1–2 cm**	Litostomatea (Ciliophora)*	40	Edgcomb and Bernhard, 2013	A, CS	>96
		Labyrinthulida (Stramenopiles)	10–20			
		Foraminifera (Rhizaria)	10–20			
	Oxic 0–1cm	Dinophysceae (Protodinium)	10–20			
		Labyrinthulida (Stramenopiles)	10–20			
Cariaco basin Permanent anoxic basin	Oxic	Radiolarians (RAD-3, RAD-19)	90	Orsi et al., 2011	CS	6489
		MAST Stramenopiles	5			
		Novel Alveolates	5			
	**Anoxic**	Radiolarians (RAD-3, RAD-19)	72			
		MAST Stramenopiles	11			
		Novel Alveolates*	14			
		Euglenozoa*	1.5			
	**Anoxic/Sulfidic**	Radiolarians (RAD-3, RAD-19)	60			
		MAST Stramenopiles	17			
		Novel Alveolates*	14			
		Euglenozoa*	9			
Indian Ocean Oxic site	Oxic Surface	Marine alveolates I	20–35	Not et al., 2008	CS	541
		Dinophysceae	10–25			
		MAST Stramenopiles	10–20			
	Oxic Chl max	Marine alveolates I	10–30			
		Radiolarians	15–40			
		MAST Stramenopiles	5–15			
Saanich Inlet Seasonally anoxic Fjord	Oxic	Stramenopiles	40–50	Orsi et al., 2012	A	4987
		Dinophysceae	20–30			
	**Suboxic**	Stramenopiles	30–40			
		Dinophysceae	30–40			
	**Anoxic**	Stramenopiles	15–25			
		Ciliophora*	15–20			
		Dinophysceae	15–20			
Saanich Inlet Seasonally anoxic	**Anoxic at 200 m**	Dinophysceae	21	Unpublished data Edgcomb	A	2000
		Dinophyta (Syndiniales)*	17			
		Stramenopiles	15			
Gulf of Mexico Seasonally anoxic	Oxic Surface	Coscinodiscophyceae	39	Rocke et al., 2012	CS	175
		Dinophysceae	40–50			
		Prasinophytes	10			
	**Anoxic**	Dinophysceae	80			
		Polycystenea	9			
		Novel Alveolates*	6			
North Pacific Coastal upwelling	Oxic Surface	Ciliophora	27	Schnetzer et al., 2011	CS	856
		Stramenopiles	20			
		Dinophysceae	14			
	**Suboxic**	Alveolate Group II*	35			
		Acantharea*	19			
		Polycystenea	16			
	**Anoxic**	Alveolate Group II*	36			
		Ciliophora*	16			
		Acantharea*	13			
Gotland Deep Seasonally anoxic	**Suboxic**	Ciliophora*	43	Stock et al., 2009	CS	600
		Fungi	30			
		Choanoflagellida	11			
	**Anoxic/Sulfidic**	Jakobida	71			
		Fungi	6			
		Ciliophora*	5			
Thetis Hypersaline anoxic basin	**Anoxic**	Fungi	37	Stock et al., 2012	CS	192
		Ciliophora*	20			
		Stramenopiles	20			
Sippewissett salt marsh	**Suboxic**	Unknown Stramenopiles	26	Stoeck et al., 2003	CS	42
		Unknown Alveolates (Ciliophora)*	26			
Black Sea Permanently anoxic	**Suboxic**	Stramenopiles	42	Wylezich and Jürgens, 2011	CS	258
		Ciliophora*	36			
		Dinoflagellates	19			
	**Anoxic/Sulfidic**	Stramenopiles	42			
		Ciliophora*	33			
		Euglenozoa*	8			
Mariajer Fjord Permanently anoxic	**Anoxic**	Alveolates (mainly Ciliophora)*	41	Zuendorf et al., 2006	CS	307
		Stramenopiles	28			
ETSP OMZ Permanently anoxic	Oxic at 5 m	Metazoa	55	This study	A	196,027
		Dinophysceae	15			
		Prymnesiophysceae	5			
	Oxic at 32 m	Dinophysceae	27			
		Dinophyta (Syndiniales)	26			
		Acantharea	17			
	Oxic at 70 m	Dinophysceae	32			
		Dinophyta (Syndiniales)	24			
		Metazoa	16			
	**Anoxic at 110 m**	Acantharea*	43			
		Dinophysceae	27			
		Dinophyta (Syndiniales)*	16			
	**Anoxic at 200 m**	Dinophysceae	40			
		Euglenozoa*	18			
		Dinophyta (Syndiniales)*	16			
	**Anoxic at 320 m**	Acantharea*	40			
		Dinophysceae	25			
		Dinophyta (Syndiniales)*	8			

a *Low oxygen samples are in bold, Oxic, O_2_ above 15 μM; Suboxic, O_2_ below 15 μM; Anoxic or Anoxic Sulfidic, no O_2_*.

b *Dominant taxa, bold font with an asterisk highlight taxa shared among multiple low oxygen sites but not common at oxic sites*.

c *Sequencing Method, CS, cloning and Sanger sequencing; A, Amplicon sequencing*.

The relationship between picoeukaryotes and oxygen in OMZs is not well understood. In contrast to the larger size fraction, the 0.2–1.6 μm fraction in the ETSP was most diverse at the OMZ core. This pattern differs from that observed in seasonally anoxic waters where microeukaryote diversity (0.22–2.7 μm) and species richness declines as oxygen decreases below 20 μM (Orsi et al., 2012). However, the presence of sequences from metazoans and larger protists (e.g., radiolarians) in the 0.2–1.6 μm fraction suggests that a portion of the DNA in this size range likely originated from lysed cells and that diversity patterns in this fraction should be interpreted cautiously. Nonetheless, depth-specific diversity trends in the picoeukaryote fraction do not directly parallel those of the larger size fraction. A similar decoupling was observed in a recent analysis of size-fractionated prokaryotic communities at this ETSP site where diversity decreased from the surface to anoxic waters in the small size fraction while increasing in the larger size fraction (Ganesh et al., 2014). Though limited to a single depth profile, our data indicate that relationships between environmental variables, notably oxygen concentration, and community diversity metrics vary depending on the subset of the community being examined, indicating that estimates of bulk eukaryote diversity and inferences of diversity-environment linkages in this system may require examination of multiple microbial size classes.

The composition of the OMZ eukaryote community in the larger size fraction was presumably strongly influenced by the community in the overlying oxic, photic zone. The majority of non-metazoan sequences in this fraction were found at the surface (5 m) and at the depth of maximum chlorophyll fluorescence (32 m), and included diverse algae, particularly photosynthetic haptophytes, cryptophytes, unaffiliated Dinophyceae, and Stramenopiles (diatoms), as well as heterotrophic radiolarians belonging to the Acantharea and putative endosymbiotic dinoflagellates of the Syndiniales. Although haptophyte and cryptophyte sequence abundance became negligible upon the transition out of the photic zone and into the OMZ, radiolarians, Dinophyceae and Syndiniales dinoflagellates remained dominant community members at all depths. The consistent signal from dinoflagellates may, to an unknown extent, reflect their presence as sinking dinospores resulting from near-surface infections, or alternatively, their presence in sinking, inactive or dead metazoan hosts. Similarly, Stramenopile sequences mostly related to photosynthetic diatoms were detectable at all depths and in both size fractions, though at low abundance (<2%). Stramenopiles, diatoms in particular, are known to form dense blooms in upwelling zones, sink rapidly when no longer growing, and are major constituents of OMZ eukaryotic communities at the surface and most likely as sinking particulate organic matter at anoxic depths (Table 2, Smetacek, 1985; Montero et al., 2007). The low relative abundance of stramenopiles in this dataset may indicate that we sampled between blooms of these taxa or during a bloom of other algal species. We cannot rule out the possibility that some of the diatom sequences recovered from anoxic depths represent active cells. It has been shown, for example, that some diatoms may respire nitrate to persist during periods of dark anoxia (Kamp et al., 2011), raising the possibility that even transient (sinking) members of the OMZ microeukaryote assemblage may contribute to bulk elemental cycling in anoxic waters.

Although broad similarities in microeukaryote composition were observed across depths, several groups were enriched within the anoxic zone. Euglenid flagellates matching most closely to *Diplonema* and ciliates of the Litostomatea increased in relative abundance in the OMZ, particularly in the larger size fraction. Diverse euglenids and ciliates are common inhabitants of anaerobic and microaerophilic marine waters (Bernard and Fenchel, 1996; Hayward et al., 2003; Orsi et al., 2011, Table 2). Clone-based molecular studies of OMZs have identified a new class of euglenids (Symbiontida) that are closely related to diplonemids, common in low oxygen waters, and appear to host sulfide-oxidizing, nitrate-reducing epibiotic bacteria (Edgcomb et al., 2010; Orsi et al., 2011, 2012). Many different ciliates, including those in the class Litostomatea, have been recovered from molecular and visual studies of low oxygen sites (Lynn, 2008; Stock et al., 2009; Edgcomb and Bernhard, 2013; Edgcomb and Pachiadaki, 2014; Pachiadaki et al., in press). Ciliates found in anaerobic habitats all have mitochondria or mitochondria-like organelles called hydrogenosomes, and pyruvate oxidation via H_2_-excretion appears to be central to their anaerobic lifestyle (Fenchel and Finlay, 1991). Taxa known to inhabit anoxic marine habitats include the karyorelictids, prostomatids, haptorids, trichostomatids, entodiniomorphids, suctorids, scuticocilliatids, heterotrichids, odontostomatids, oligotrichids, and hypotrichids, some of which may be facultative anaerobes (Corliss, 1979; Fenchel and Finlay, 1995). Ciliates are often important grazers of abundant prokaryotes found along oxyclines, including in the deep sea (e.g., Lin et al., 2008; Pachiadaki et al., in press), and hence, may contribute significantly to nutrient cycling along OMZ oxygen gradients. *Perispira ovum*, a litostomatean ciliate, has been shown to prey heavily on euglenids, sequestering their chloroplasts and mitochondria as a potential mechanism to cope with low oxygen, thus acquiring the ability to function as mixotrophs in environments where mixotrophy confers a competitive advantage (Johnson et al., 1995). The lifestyle of litostomatean ciliates in the core of this OMZ, well outside of the photic zone, can only be speculated upon, and if these ciliates are active, other adaptive mechanisms may be present within taxa not normally described from dark, anoxic, deep water columns. Within both of these diverse groups (ciliates and euglenids detected in this study), physiological adaptions to oxygen depletion and mutualistic associations with prokaryotes may enable persistence in OMZs.

Dinoflagellates of the order Syndiniales showed clear depth-specific differences. This group predominated within the 0.2–1.6 μm size fraction, increasing in richness at the OMZ core and contributing to an overall peak in diversity at this depth (Figures 1, 2E). The Syndiniales are thought to be exclusive endoparasites of macrozoans and other protists (Moreira and López-García, 2003; Chambouvet et al., 2008) and are frequently reported as the most abundant picoeukaryotes in environmental clone libraries from diverse ocean sites (López-García et al., 2001; Countway et al., 2007; Not et al., 2007; Sauvadet et al., 2010). Syndiniales are known to play important roles in controlling blooms of different taxa, and these parasitoids complete their life cycle in less than 3 days, releasing hundreds of free-living dinospores (<2 μm) in the process (Chambouvet et al., 2008; Guillou et al., 2008). The abundance of dinospores may contribute to dominance of Syndiniales in many environmental surveys. Although the diversity and environmental distribution of Syndiniales are not fully understood, prior evidence shows that Groups I and II are present at hypoxic and suboxic sites (Guillou et al., 2008), with Group II more common than Group I below the photic zone. Here, Syndiniales Group II clades 5, 21, and 30 increased in relative abundance within the ETSP OMZ compared to more oxygenated waters above and below. Conversely, Clade 7 (Group II), the dominant Syndiniales group within the oxycline and in the oxic depths, decreased in abundance at the OMZ core. Some Group II clades (specifically 1, 2, and 14) are parasites of other dinoflagellates and appear to show host specificity (Chambouvet et al., 2008). It is unclear whether the vertical distribution and ecological contributions of these and other Syndiniales clades in the ETSP OMZ are driven by free-living Syndiniales cells or by associations with hosts whose distributions vary with depth (assuming host lysis and release of cells into the picoeukaryotic fraction). Indeed, clone libraries from anoxic waters appear to contain members of the Dinophysceae sharing low similarity to known dinoflagellates who may serve as hosts to Syndiniales (Stoeck et al., 2003; Wylezich and Jürgens, 2011).

Radiolarian protists related to *Acantharea* were also enriched in the OMZ. This may suggest they are also transported to the OMZ interior as sinking cells from the surface, potentially as aggregates or in association with fecal pellets (Turner, 2002). However, sequences affiliating with Radiolaria are frequently extremely abundant in environmental surveys of oxygen-depleted water columns (e.g., Not et al., 2008; Countway et al., 2010; Edgcomb et al., 2011a; Table 2). In the ETSP, acantharids peaked in proportional abundance at the upper portion of the OMZ (110 m) and again at 320 m in the anoxic core. Acantharids are mixotrophs that are common in surface waters, especially in eutrophic environments (Gilg et al., 2010), where they have been shown to host large numbers of photosynthetic eukaryotic symbionts (Michaels, 1991; Michaels et al., 1995). They are thought to be the most significant biological regulators of strontium in the ocean as they construct their cell walls entirely of strontium sulfate. Interestingly, these taxa are in very low abundance (<3%) at 200 m and at intermediate abundance (14–15%) in the oxic 32 and 70 m samples. The acantharid-affiliated OTUs that were abundant in the OMZ at 110 and 320 m were also present at surface depths, suggesting the transport of these taxa into the OMZ via sinking particles. However, we did not observe an even distribution of these signatures at depths between the surface and 320 m, and so the patchy distribution of this group is inconsistent with a uniform flux of dead or dying radiolarians from the photic zone. Measurements of absolute abundance along with indicators of metabolic activity would be required to confirm and interpret this pattern.

Over half of all sequences at the surface (>1.6 μm fraction) were affiliated with metazoans (Figures 2A,D). While the dominant urochordate signal detected at the surface was lost in the transition into the OMZ, cnidarian, ctenophore, and *Maxillipoda* (copepod)- associated sequences detected at the surface were present in anoxic depths at low abundance (<10% of total sequences). Sequences matching jellyfish (Cnidaria) dominated the metazoan sequence pool in the anoxic zone. Diverse jellyfish have been observed in the oxycline and upper hypoxic layers of the OMZ of the Eastern Tropical North Pacific (Beatteay, 2012, unpublished), consistent with the observed tolerance of many jellyfish to hypoxia (Elliott et al., 2012). Copepods have also been observed in diverse low oxygen waters where they exhibit species-specific depth distributions (Saltzman and Wishner, 1997) and may possess physiological adaptations such as reduced respiration that allow persistence for brief periods under hypoxia (Teuber et al., 2013). Here, sequences matching copepods increased in relative abundance from the oxycline to the anoxic OMZ core before declining again at 1000 m, raising the potential that these organisms may occupy a niche within the anoxic zone where they may escape predation or have less competition. Additional taxonomic and physiological characterizations are needed to confirm the presence of a metazoan community adapted specifically to the extreme oxygen depletion observed in the ETSP.

### Comparisons to other low oxygen sites

The taxonomic trends in the ETSP show broad similarities to those observed at other low oxygen marine sites, as well as notable differences, which may reflect the unique habitat characteristics of an OMZ. The relative dominance of dinoflagellate and radiolarian signatures in the ETSP OMZ has been observed at several other oxygen-depleted sites in upwelling regions, including the Black Sea, Mariager Fjord, North Pacific, Saanich Inlet, and the Gulf of Mexico (Table 2), although due to potentially high ribosomal RNA gene copy numbers in these groups, these observations must be interpreted with caution until they are confirmed by microscopy or another method. Signatures of ciliates and euglenids were more abundant in our libraries from within the OMZ core and are also common in molecular surveys from other low oxygen environments. For example, litostomatean ciliates were identified in an Eastern Mediterranean anoxic basin (Edgcomb and Bernhard, 2013) and signatures of other diverse ciliates, including novel lineages have been recovered from Mariager fjord, the Black Sea, Baltic Sea, and the North Pacific (Table 2). Euglenid signatures were abundant in molecular surveys of both the Black Sea and Cariaco Basin (Table 2). Stramenopiles appear to be common across anoxic environments, but were rarely detected in our ETSP OMZ dataset.

The sequences recovered from many of these “low oxygen” groups vary by region and often do not match known sequences in public databases that would enable finer taxonomic assignments (Orsi et al., 2012; Stock et al., 2012; Edgcomb and Bernhard, 2013). This highlights a general gap in knowledge of the underlying diversity in oxygen-depleted waters. Furthermore, studies of eukaryote diversity in low oxygen waters have utilized an array of sampling methods, sample sizes, and analysis tools, making it challenging to directly compare diversity metrics across sites (Stock et al., 2009; Edgcomb et al., 2011a; Orsi et al., 2011; Rocke et al., 2012). For example, the analysis of multiple size fractions in our study identifies contrasting patterns of phylogenetic diversity with depth in the OMZ, suggesting knowledge of the effect of environmental gradients (e.g., oxygen) on community structure may be biased depending on sample collection and processing strategy. Together, the increasing volume of sequence data from diverse low oxygen regions suggests geographic differences in protist communities in different oxygen-poor habitats, and high levels of uncharacterized microeukaryote diversity (Stoeck et al., 2003; Zuendorf et al., 2006; Not et al., 2008; Schnetzer et al., 2011; Orsi et al., 2012). Resolving linkages between environmental parameters and diversity of microeukaryotes in OMZs will require application of uniform sampling methods across diverse low oxygen waters, as well as studies that couple gene surveys to genomic or ecophysiological measurements to link OTU distributions to functional activity.

### Conflict of interest statement

The authors declare that the research was conducted in the absence of any commercial or financial relationships that could be construed as a potential conflict of interest.

## References

[B72] Arístegui J. Gasol J. M. Duarte C. M. Herndl G. J. (2009). Microbial oceanography of the dark ocean's pelagic realm. Limnol. Oceanogr. 54, 1501–1529. 10.4319/lo.2009.54.5.1501

[B1] Beatteay S. L. (2012). Impact of Hypoxia on the Vertical Distribution of Jellyfish in the Eastern Tropical Northern Pacific. University of Washington Senior Thesis.

[B2] Bernard C. Fenchel T. (1996). Some microaerobic ciliates are facultative anaerobes. Eur. J. Protistol. 32, 293–297. 10.1016/S0932-4739(96)80051-4

[B3] Bernhard J. M. Buck K. R. Farmer M. A. Bowser S. S. (2000). The Santa Barbara Basin is a symbiosis oasis. Nature 403, 77–80. 10.1038/47476 10638755

[B73] Bråte J. Logares R. Berney C. Ree D. K. Klaveness D. Jakobsen K. S.. (2010). Freshwater Perkinsea and marine-freshwater colonizations revealed by pyrosequencing and phylogeny of environmental rDNA. ISME J. 4, 1144–1153. 10.1038/ismej.2010.39 20393574

[B4] Caporaso J. Kuczynski J. Stombaugh J. Bittinger K. Bushman F. D. Costello E. K.. (2010). QIIME allows analysis of high-throughput community sequencing data. Nat. Methods 7, 335–336. 10.1038/nmeth.f.303 20383131 PMC3156573

[B5] Chambouvet A. Morin P. Marie D. Guillou L. (2008). Control of toxic marine dinoflagellate blooms by serial parasitic killers. Science 322, 1254–1257. 10.1126/science.1164387 19023082

[B71] Childress J. J. Seibel B. A. (1998). Life at stable low oxygen levels: adaptations of animals to oceanic oxygen minimum layers. J. Exp. Biol. 201, 1223–1232. 9510533 10.1242/jeb.201.8.1223

[B6] Corliss J. O. (1979). The Ciliated Protozoa. Oxford: Pergamon Press

[B7] Countway P. D. Gast R. J. Dennett M. R. Savai P. Rose J. M. Caron D. A. (2007). Distinct protistan assemblages characerize the euphotic zone and deep sea (2500 m) of the western North Atlantic (Sargasso Sea and Gulf Stream). Environ. Microbiol. 9, 1219–1232. 10.1111/j.1462-2920.2007.01243.x 17472636

[B8] Countway P. D. Vigil P. D. Schnetzer A. Moorthi S. D. Caron D. A. (2010). Seasonal analysis of protistan community structure and diversity at the USC Microbial Observatory (San Pedro Channel, North Pacific Ocean). Limnol. Oceanogr. 55, 2381–2396. 10.4319/lo.2010.55.6.2381

[B9] Diez B. Pedros-Alio C. Massan R. (2001). Study of genetic diver-sity of eukaryotic picoplankton in different oceanic regions by small-subunit rRNA gene cloning and sequencing. Appl. Environ. Microbiol. 67, 2932–2941. 10.1128/AEM.67.7.2932-2941.2001 11425705 PMC92964

[B11] Edgcomb V. Bernhard J. (2013). Heterotrophic protists in hypersaline microbial mats and deep hypersaline basin water columns. Life 3, 346–362. 10.3390/life3020346 PMC4187137 25369746

[B12] Edgcomb V. Breglia S. A. Yubuki N. Beaudoin D. Patterson D. J. Leander B. S.. (2010). Identity of epibiotic bacteria on symbiontid euglenozoans in O_2_-depleted marine sediments: evidence for symbiont and host co-evolution. ISME J. 5, 1–13. 10.1038/ismej.2010.121 20686514 PMC3105687

[B14] Edgcomb V. P. Orsi W. Breiner H.-W. Stock A. Filker S. Yakimov M. M.. (2011b). Novel kinetoplastids associated with hypersaline anoxic lakes in the Eastern Mediterranean deep-sea. Deep Sea Res. I 58, 1040–1048. 10.1016/j.dsr.2011.07.00322817877

[B10] Edgcomb V. Orsi W. Bunge J. Jeon S. Christen R. Leslin C.. (2011a). Protistan microbial observatory in the Cariaco Basin, Caribbean. I. Pyrosequencing vs Sanger insights into species richness. ISME J. 5, 1344–1356. 10.1038/ismej.2011.6 21390079 PMC3146274

[B13] Edgcomb V. P. Pachiadaki M. (2014). Ciliates along oxyclines of permanently stratified marine water columns. J. Eukaryot. Microbiol. 4, 434–445. 10.1111/jeu.12122 24801774

[B15] Elliott D. T. Pierson J. Roman M. (2012). Relationship between environmental conditions and zooplankton community structure during summer hypoxia in the northern Gulf of Mexico. J. Plankton Res. 34, 443–453. 10.1093/plankt/fbs029

[B16] Faith D. P. (1992). Conservation evaluation and phylo- genetic diversity. Biol. Conserv. 61, 1–10. 10.1016/0006-3207(92)91201-3

[B17] Fenchel T. Finlay B. J. (1991). The biology of free-living anaerobic ciliates. Eur. J. Protistol. 26, 201–215. 10.1016/S0932-4739(11)80143-4 23196279

[B18] Fenchel T. Finlay B. J. (1995). Ecology and evolution in anoxic worlds, oxford series, in Ecology and Evolution, eds May R. M. Harvey P. H. (New York, NY: Oxford University Press), 276.

[B19] Fogg G. E. (1995). Some comments on picoplankton and its importance in the pelagic ecosystem. Aquat. Microbial. Ecol. 9, 33–39. 10.3354/ame009033

[B20] Ganesh S. Parris D. J. DeLong E. F. Stewart F. J. (2014). Metagenomic analysis of size-fractionated picoplankton in a marine oxygen minimum zone. ISME J. 8, 187–211. 10.1038/ismej.2013.144 24030599 PMC3869020

[B21] Gilg I. Amaral-Zettler L. Countway P. Moorthi S. Schnetzer A. Caron D. (2010). Phylogenetic affiliations of mesopelagic acantharia and acantharian-like environmental 18S rRNA genes off the southern California coast. Protist 161, 197–211. 10.1016/j.protis.2009.09.002 20044311

[B22] Grossart H. P. Hietanen S. Ploug H. (2003). Microbial dynamics on diatom aggregates in Øresund, Denmark. Mar. Ecol. Prog. Ser. 249, 69–78. 10.3354/meps249069

[B24] Guillou L. Bachar D. Audic S. Bass D. Berney C. Bittner L.. (2012). The Protist Ribosomal Reference database (PR2): a catalog of unicellular eukaryote small sub-unit rRNA sequences with curated taxonomy. Nucleic Acids Res. 41, D597–D604. 10.1093/nar/gks1160 23193267 PMC3531120

[B23] Guillou L. Viprey M. Chambouvet A. Welsh R. Kirkham A. Massana R. (2008). Widespread occurrence and genetic diversity of marine parasitoids belonging to Syndiniales (Alveolata). Environ. Microbiol. 10, 3349–3365. 10.1111/j.1462-2920.2008.01731.x 18771501

[B25] Hayward B. H. Drosteb R. Epstein S. S. (2003). Interstitial ciliates: benthic microaerophiles or planktonic anaerobes? J. Eukaryot. Microbiol. 50, 356-359. 10.1111/j.1550-7408.2003.tb00148.x 14563174

[B26] Johnson P. W. Donaghay P. L. Small E. B. Sieburth J. M. (1995). Ultrastructure and ecology of Perispira ovum (Ciliophora:Litostomatea): an aerobic, planktonic ciliate that sequesters the chloroplasts, mitochondria, and paramylon of Euglena proxima in a micro-oxic habitat. Eukaryot. Microbiol. 42, 323–335. 10.1111/j.1550-7408.1995.tb01588.x

[B27] Kamp A. de Beer D. Nitsch J. L. Lavik G. Stief P. (2011). Diatoms respire nitrate to survive dark and anoxic conditions. Proc. Natl. Acad. Sci. U.S.A. 108, 5649–5654. 10.1073/pnas.1015744108 21402908 PMC3078364

[B28] Lam P. Lavik G. Jensen M. Vossenberg J. Schmid M. Woebken D.. (2009). Revising the nitrogen cycle in the Peruvian oxygen minimum zone. Proc. Natl. Acad. Sci. U.S.A. 106, 4752–4757. 10.1073/pnas.0812444106 19255441 PMC2649953

[B29] Levin L. A. (2003). Oxygen minimum zone benthos: adaptation and community response to hypoxia. Oceanogr. Marine Biol. 41, 1–45

[B30] Li X. N. Taylor G. T. Astor Y. Scranton M. I. (2008). Sulfur speciation in the Cariaco Basin with reference to chemoautotrophic production. Mar. Chem. 112, 53–64. 10.1016/j.marchem.2008.06.002

[B31] Lin X. J. Scranton M. I. Chistoserdov A. Varela R. Taylor G. T. (2008). Spatiotemporal dynamics of bacterial populations in the anoxic Cariaco Basin. Limnol Oceanogr. 53, 37–51. 10.4319/lo.2008.53.1.0037

[B32] López-García P. Rodríguez-Valera F. Pedrós-Alió C. Moreira D. (2001). Unexpected diversity of small eukaryotes in deep-sea Antarctic plankton. Nature 409, 603–607. 10.1038/35054537 11214316

[B33] Lozupone C. Knight R. (2005). UniFrac: a new phylogenetic method for comparing microbial communities. Appl. Environ Microbiol. 71, 8228–8235. 10.1128/AEM.71.12.8228-8235.2005 16332807 PMC1317376

[B34] Lynn D. (2008). The Ciliated Protozoa: Characterization, Classification, and Guide to the Literature. 3rd Edn. New York, NY: Springer

[B35] Massana R. Pernice M. Bunge J. A. Del Campo J. (2011). Sequence diversity and novelty of natural assemblages of picoeukaryotes from the Indian Ocean. ISME J. 5, 184–195. 10.1038/ismej.2010.104 20631807 PMC3105688

[B36] Michaels A. F. (1991). Acantharian abundance and symbiont productivity at the VERTEX seasonal station. J. Plankton Res. 13, 399–418. 10.1093/plankt/13.2.399

[B37] Michaels A. F. Caron D. A. Swanberg N. R. Howse F. A. Michaels C. M. (1995). Planktonic sarcodines (acantharia, radiolaria, foraminifera) in surface waters near Bermuda: Abundance, biomass and vertical flux. J. Plankton Res. 17, 131–163

[B38] Montero M. Daneri G. Cuevas L. A. González H. E. Jacob B. Lizárraga L.. (2007). Productivity cycles in the coastal upwelling area off Concepcion: the importance of diatoms and bacterioplankton in the organic carbon flux. Prog. Oceanogr. 75, 518–530. 10.1016/j.pocean.2007.08.013

[B39] Moon-van der Staay S. Y. De Wachter R. Vaulot D. (2001). Oceanic 18S rDNA sequences from picoplankton reveal new eukaryotic lineages. Nature 409, 607–610. 10.1038/35054541 11214317

[B40] Moreira D. López-García P. (2003). Are hydrothermal vents oases for parasitic protists? Trends Parasitol. 19, 556–558. 10.1016/j.pt.2003.09.013 14642764

[B41] Neira C. Sellanes J. Levin L. A. Arntz W. E. (2001). Meiofaunal distributions on the Peru margin: relationship to oxygen and organic matter availability. Deep-Sea Res. 48, 2453–2472. 10.1016/S0967-0637(01)00018-8

[B42] Not F. Gausling R. Azam F. Heidelberg J. F. Worden A. Z. (2007). Vertical distribution of picoeukaryotic diversity in the Sargasso Sea. Environ. Microbiol. 9, 1233–1252. 10.1111/j.1462-2920.2007.01247.x 17472637

[B43] Not F. Latasa M. Scharek R. Viprey M. Karleskind P.. (2008). Phytoplankton diversity across the Indian Ocean: a focus on the picoplanktonic size fraction. Deep-Sea Res. I 55, 1456–1473. 10.1016/j.dsr.2008.06.007

[B44] Orsi W. Edgcomb V. (2013). Microbial eukaryotes in marine oxygen minimum zones. Polyextremophiles 27, 485–497. 10.1007/978-94-007-6488-0_21

[B45] Orsi W. Edgcomb V. Jeon S. Leslin C. Bunge J. Taylor G. T.. (2011). Protistan microbial observatory in the Cariaco Basin, Caribbean. II. Habitat specialization. ISME J. 5, 1357–1373. 10.1038/ismej.2011.7 21390077 PMC3146276

[B46] Orsi W. Song Y. C. Hallam S. Edgcomb V. (2012). Effect of oxygen minimum zone formation on communities of marine protists. ISME J. 6, 1586–15601. 10.1038/ismej.2012.7 22402396 PMC3400406

[B47] Pachiadaki M. Taylor C. Oikomomou A. Yakimov M. Stoeck T. Edgcomb V. P. (in press). Grazing studies conducted *in situ* reveal protist turnover of bacterial biomass in the Deep E. Mediterranean Sea. Deep Sea Res. II.

[B48] Revsbech N. P. Larsen L. H. Gundersen J. K. Dalsgaard T. Ulloa O. Thamdrup B. (2009). Determination of ultra-low oxygen concentrations in oxygen minimum zones by the STOX sensor. Limnol. Oceanogr. Methods 7, 371–381. 10.4319/lom.2009.7.371

[B49] Rocke E. Hongmei J. Liu H. (2012). Phylogenetic composition and distribution of picoeukaryotes in the hypoxic northwestern coast of the Gulf of Mexico. Microbiologyopen 2, 1, 130–143. 10.1002/mbo3.57 23281331 PMC3584219

[B50] Saltzman J. Wishner K. F. (1997). Zooplankton ecology in the eastern Pacific oxygen minimum zone above a seamount: 2. Vertical distribution of copepods. Deep-Sea Res. I 44, 931–954. 10.1016/S0967-0637(97)00006-X

[B51] Sauvadet A.-L. Gobet A. Guillou L. (2010). Comparative analysis between protist communities from the deep-sea pelagic ecosystem and specific deep- hydrothermal habitats. Environ. Microbiol. 12, 2946–2964. 10.1111/j.1462-2920.2010.02272.x 20561018

[B52] Schnetzer A. Moorthi S. Countway P. Gast R. Gilg I. Caron D. (2011). Depth matters: microbial eukaryote diversity and community structure in the eastern North Pacific revealed through environmental gene libraries. Deep Sea Res. I 58, 16–26. 10.1016/j.dsr.2010.10.003

[B74] Sherr E. B. Sherr B. F. (2002). Significance of predation by protists in aquatic microbial food webs. Antonie Leeuwenhoek Int. J. Gen. Mol. Microbiol. 81, 293–308. 10.1023/A:1020591307260 12448728

[B53] Smetacek V. S. (1985). Role of sinking in diatom life-history cycles: ecological, evolutionary, and geological significance. Marine Biol. 84, 239–251. 10.1007/BF00392493

[B54] Stevens H. Ulloa O. (2008). Bacterial diversity in the oxygen minimum zone of the eastern tropical South Pacific. Environ. Microbiol. 10, 1244–1259. 10.1111/j.1462-2920.2007.01539.x 18294206

[B55] Stock A. Breiner H. W. Pachiadaki M. Edgcomb V. Filker S.. (2012). Microbial eukaryote life in the new hypersaline deep-sea basin Thetis. Extremophiles 16, 21–34. 10.1007/s00792-011-0401-4 22009262

[B56] Stock A. Bunge J. Jürgens K. Stoeck T. (2009). Protistan diversity in the suboxic and anoxic waters of the Gotland Deep (Baltic Sea) as revealed by 18S rRNA clone libraries. Aquat. Microb. Ecol. 55, 267–284. 10.3354/ame01301

[B57] Stoeck T. Taylor G. T. Epstein S. S. (2003). Novel eukaryotes from a permanently anoxic Cariaco Basin (Caribbean Sea). Appl. Environ. Microbiol 69, 5656–5663. 10.1128/AEM.69.9.5656-5663.2003 12957957 PMC194984

[B58] Tang K. W. Turk V. Grossart H. P. (2010). Linkage between crustacean zooplankton and aquatic bacteria. Aquat. Microb. Ecol. 61, 261–277. 10.3354/ame01424

[B59] Taylor G. T. (1982). The role of pelagic heterotrophic protozoa in nutrient cycling: a review. Ann. Inst. Oceanogr. Paris 58, 227–241

[B60] Taylor G. T. Scranton M. I. Iabichella M. Ho T. Thunell R. C. Muller-Karger F.. (2001). Chemoautotrophy in the redox transition zone of the Cariaco Basin: a significant midwater source of organic carbon production. Limnol. Oceanogr. 46, 148–163. 10.4319/lo.2001.46.1.0148

[B61] Teuber L. Kiko R. Séguin F. Auel H. (2013). Respiration rates of tropical Atlantic copepods in relation to the oxygen minimum zone. J. Exp. Mar. Biol. Ecol. 448, 28–36. 10.1016/j.jembe.2013.06.01224223716

[B62] Thamdrup B. Dalsgaard T. Jensen M. M. Ulloa O. Farías L. Escribano R. (2006). Limnol. Oceanogr. 51, 2145–2156. 10.4319/lo.2006.51.5.2145

[B63] Thamdrup B. Dalsgaard T. Revsbech N. P. (2012). Widespread functional anoxia in the oxygen minimum zone of the eastern South Pacific. Deep Sea Res. 65, 36–45. 10.1016/j.dsr.2012.03.00123766277

[B64] Turner J. T. (2002). Zooplankton fecal pellets, marine snow and sinking phytoplankton blooms. Aquat. Microb. Ecol. 27, 57–102. 10.3354/ame027057

[B65] Ulloa O. Canfield D. E. Delong E. F. Letelier R. M. Stewart F. J. (2012). Microbial oceanography of anoxic oxygen minimum zones. Proc. Natl. Acad. Sci. U.S.A. 109, 15996–16003. 10.1073/pnas.1205009109 22967509 PMC3479542

[B66] Worden A. Z. Nolan J. K. Palenik B. (2004). Assessing the dynamics and ecology of marine picophytoplankton: The importance of the eukaryotic component. Limnol. Oceanogr. 49, 168–179. 10.4319/lo.2004.49.1.0168

[B67] Wright J. J. Konwar K. M. Hallam S. J. (2012). Microbial ecology of expanding oxygen minimum zones. Nat. Rev. Microbiol. 10, 381–394. 10.1038/nrmicro2778 22580367

[B68] Wylezich C. Jürgens K. (2011). Protist diversity in suboxic and sulfidic waters of the Black Sea. Environ. Microbiol. 13, 2939–2956. 10.1111/j.1462-2920.2011.02569.x 21951296

[B69] Yubuki N. Edgcomb V. P. Bernhard J. M. Leander B. S. (2009). Ultrastructure and molecular phylogeny of Calkinsia aureus: cellular identity of a novel clade of deep-sea euglenozoans with epibiotic bacteria. BMC Microbiol. 9:16. 10.1186/1471-2180-9-16 19173734 PMC2656514

[B70] Zuendorf A. Bunge J. Behnke A. Barger K. J. A. Stoeck T. (2006). Diversity estimates of microeukaryotes below the chemocline of the anoxic Mariager Fjord, Denmark. Fems Microbiol Ecol 58, 476–491. 10.1111/j.1574-6941.2006.00171.x 17117990

